# Association of *ERAP1* and *ERAP2* gene polymorphisms and ERAP2 protein with the susceptibility and severity of rheumatoid arthritis in the Ukrainian population

**DOI:** 10.3389/fimmu.2024.1519159

**Published:** 2025-01-21

**Authors:** Iryna Kril, Andrzej Wiśniewski, Agnieszka Tarnowska, Khrystyna Lishchuk-Yakymovych, Yaryna Bojko, Piotr Kuśnierczyk, Valentyna V. Chopyak, Izabela Nowak

**Affiliations:** ^1^ Department of Clinical Immunology and Allergology, Danylo Halytsky Lviv National Medical University, Lviv, Ukraine; ^2^ Laboratory of Immunogenetics and Tissue Immunology, Hirszfeld Institute of Immunology and Experimental Therapy Polish Academy of Sciences, Wrocław, Poland

**Keywords:** rheumatoid arthritis, MHC I class, ERAP1, ERAP2, single nucleotide polymorphisms

## Abstract

**Introduction:**

Rheumatoid arthritis (RA) is a long-term autoimmune disorder that primarily affects joints. Although RA is chiefly associated with HLA class II, nevertheless some HLA class I associations have also been observed. These molecules present antigenic peptides to CD8+ T lymphocytes and natural killer cells. HLA-I molecules bind their peptide cargo (8–10 amino acids long) in the endoplasmic reticulum. Peptides longer than 10 amino acids are trimmed by the endoplasmic reticulum aminopeptidases ERAP1 and ERAP2 to fit the peptide binding groove of the HLA-I molecule. Here, we investigated the possible association of *ERAP1* and *ERAP2* polymorphisms with RA, and also any possible correlation between serum levels of the ERAP2 protein with disease severity.

**Methods:**

We used Real-Time PCR to genotype *ERAP1* and *ERAP2* and ELISA test to detect *ERAP2* protein.

**Results:**

We found significant associations of *ERAP1* rs30187, rs27044, and rs26618, as well as *ERAP2* rs2248374, with susceptibility to RA. *ERAP1* rs26653 and ERAP2 rs2248374 were also associated with the Disease Activity Score (DAS28), and some polymorphisms were also associated with anti-citrullinated protein or anti-mutated citrullinated vimentin antibodies. RA patients secreted higher concentrations of ERAP2 than controls. Patients with mild disease activity (DAS28 < 3.2) released a concentration of ERAP2 four times lower than that of patients with severe disease activity (DAS28 > 5.1). We detected a higher level of *ERAP2* in rheumatoid factor (RF)-positive patients than in RF-negative patients. *ERAP2* concentration above 5.85 ng/mL indicated a severe phase of RA.

**Conclusions:**

Some *ERAP1* and *ERAP2* polymorphisms seem to be related to susceptibility to RA or the severity of the disease. The ERAP2 protein tested in serum could be a valuable biomarker of RA severity.

## 1 Introduction

Rheumatoid arthritis (RA) is a long-term autoimmune inflammatory disease affecting the joints, connective tissue and organs, manifesting in joint pain, swelling, stiffness, and loss of joint function (1, 2). Multiple factors influence RA development, including genetic predisposition and various environmental and lifestyle elements. It has been shown that women are more likely to suffer from RA, and the ratio of women to men is approximately 3:1 (3). The heredity of RA is around 65%, which emphasizes the importance of genetics (4). Among genetic factors, various genes have been associated with RA predisposition. Genetic association studies on several different populations have identified 150 candidate loci with polymorphisms connected to RA, primarily related to seropositive disease and HLA associations (5, 6).

During the autoimmune process, immune responses are triggered against peptides presented in the context of Major Histocompatibility Complex (MHC) class I or class II molecules, referred to as HLA (human leukocyte antigen) in humans. Although rheumatoid arthritis is primarily associated with HLA class II (7, 8), nevertheless some HLA class I associations have also been observed (9, 10), as supported by the association of some *KIR* genes (11–13) whose products, killer cell immunoglobulin-like receptors, recognize an absence or decrease in the expression of MHC class I molecules on the surface of target cells. MHC-I-bound peptides influence this interaction. KIR molecules are expressed on NK cells and subpopulations of T lymphocytes (14) which could be engaged in joint inflammation and destruction.

Antigenic peptides are formed and loaded on MHC class I molecules in the endoplasmic reticulum (ER) during a multistep process involving many proteins collectively named antigen processing and presentation machinery (APM) (15). Important elements of the APM are the endoplasmic reticulum aminopeptidases ERAP1 and ERAP2. ERAP1 and the closely related ERAP2 belong to the M1 family of zinc-metallopeptidase enzymes. Both aminopeptidases are IFN-γ and TNF-α inducible and ubiquitously expressed. They share ~ 49% sequence similarity and can form functional heterodimers (16–18). They are assigned to produce optimal-sized peptides for HLA class I binding by deleting amino acids from the unblocked N-terminus of peptides. Trimmed peptides with 8-10 amino acids can be directly presented by MHC class I molecules on the cell surface (19).

*ERAP1* and *ERAP2* single nucleotide polymorphisms (SNPs) affect both activity and specificity of the enzymes. Therefore, *ERAP1* SNPs have been correlated with HLA-associated diseases like ankylosing spondylitis (AS) (20), psoriasis (21, 22), type 1 diabetes (T1D) (23), inflammatory bowel disease (24), Behçet’s disease (25), cervical cancer (26), and hypertension (27), suggesting that *ERAP1* polymorphisms can exert diverse effects. Numerous literature data indicate that the *ERAP1* and *ERAP2* SNPs may not only affect the correct trimming and presentation of antigenic peptides to the respective receptors in the context of HLA class I, but also actively modulate peptide diversity (28, 29).

The aim of this work was to examine the role of *ERAP1/ERAP2* polymorphisms in the susceptibility to RA and the severity of this disease. Moreover, we wanted to check the impact of the ERAP2 protein tested in the serum of patients with clinical manifestations. This work is the first report presenting the association of ERAP2-secreted protein with RA.

## 2 Materials and methods

### 2.1 Study design

Two hundred and ninety-five RA patients (103 men and 192 women) and 237 healthy controls (112 men and 125 women) were qualified for the study. All tested participants were of Ukrainian origin. Of this group, 72 were children diagnosed with juvenile-onset arthritis (JIA) and belonged to three subtypes of JIA that can probably transform into RA. Eighty-six percent of these patients for prevention of active clinical, and laboratory progression of possible RA are still on immunobiological therapy and responded positively and don’t respond effectively to non-steroidal anti-inflammatory drugs (NSAIDs). Without immunobiological therapy, they have active arthritis symptoms and joint dysfunction because the possibility of future RA development is at a high level. Ten percent of these patients have active joint and myalgic syndrome but without positive anti-cyclic citrullinated peptide (anti-CCP) antibodies.

This study was carried out according to the Declaration of Helsinki and accepted by the Ethics Committee of Danylo Halytsky Lviv National Medical University (protocol No. 5, from 22 June 2020). Additional consent to continue the research study in Poland was obtained at the Hirszfeld Institute of Immunology and Experimental Therapy by the Bioethics Commission on 11 June 2022 (No: KB -7/2022). All RA patients met the American College of Rheumatology (ACR)/European League Against Rheumatism (EULAR) criteria for RA (30). The activity of rheumatoid arthritis in patients was assessed using the Disease Activity Score 28 (DAS28), which included: the number of tender joints (0-28), the number of swollen joints (0-28), CRP (1.0-100 mg/l) and the patient’s assessment of the general health status based on a 10-point visual analog scale. In 2021 and 2022, RA patients were treated in the Lviv Regional Clinical Hospital’s Rheumatology Department and the Regional Center of Clinical Immunology and Allergology outpatient clinics. The control group consisted of 231 blood donors from the Lviv Regional Blood Donation and Blood Treatment Center [all with negative rheumatoid factor (RF)] and 6 healthy boys aged 16 and 17 years, the control group in another study. It should be emphasized that the parents gave their consent to have their blood taken for our study. All cases and controls signed written informed consent to participate in the study. **Table 1** presents the clinical characteristics of all participants of the research.

**Table 1:** RA patients and control group demographic and clinical characteristics.

	Patients			Control		
	N	Female	Male	N	Female	Male
All	295	192 (65.08)	103 (34.92)	237	125 (52.74)	112 (47.26)
Adults	223 (75.59)	159 (71.30)	64 (28.70)	231 (97.47)	125 (54.11)	106 (45.89)
Children	72 (24.41)	33 (45.83)	39 (54.17)	6 (2.53)	0 (0.00)	6 (100.00)
	N	Range	Mean ± SD		Range	Mean ± SD
Age [years]		2-88	36.11 ± 18.36		16-64	30.77 ± 9.34
RA duration [years]		0.5-54	10.95 ± 10.15			
Family RA story	70 (23.73)					
Joint pain	264 (89.49)					
Joint swelling	216 (73.22)					
Muscle pain	107 (36.20)					
RF (+)	151 (51.01)	0-48	11 ± 11			
Anti-CCP (+)	174 (58.58)	1-265	74.09 ± 62.65			
Anti-MCV (+)	151 (51.01)	20-41	28 ± 4.99			
ANA	128 (43.39)	1.2–10.11	3.79 ± 2.01			
ESR (+)	219 (74.23)	5-61	26 ± 10.01			
CRP (+)	169 (57.29)	0-120	23 ± 21			
Glucocorticosteroids	242 (82.03)					
Methotrexate	124 (42.03)					
Biological therapy	94 (31.86)					

RF, rheumatoid factor; Anti-CCP, anti-citrullinated protein; Anti-MCV, anti-mutated citrullinated vimentin; ANA, anti-nuclear antibodies; ESR, erythrocyte sedimentation rate (positive ESR results were assumed to be >15 mm/h for men and >20 mm/h for women obtained using the Westergren method); CRP, C-reactive protein. Values in parentheses are in percentages.

### 2.2 DNA preparation and SNP genotyping

Genomic DNA was isolated from refrozen venous blood using the NucleoSpin^®^ Blood mini kit (Macherey-Nagel, Germany) according to the manufacturer’s protocol. Five SNPs in *ERAP1* (rs26653, rs26618, rs30187, rs27044, rs7063) and one in *ERAP2* (rs2248374) were genotyped using the TaqMan SNP Genotyping Assays (Applied Biosystems, Foster City, USA) according to the manufacturer’s instructions. 7300 Real-Time PCR System and SDS software ver. 1.4 (Applied Biosystems, Foster City, USA) was used to carry out the PCR reactions and allelic discrimination, respectively. Characteristics of the *ERAP1/2* SNPs examined in this study are shown in **Table 2**.

**Table 2:** Characteristics of the SNPs examined in this study.

Gene, locus	SNP region	SNP variation	Biological effect	Assay ID
ERAP1 5q15	rs26618 exon 5	C > T, M276I	aminopeptidase activity (able to efficiently generate the precursor of the HLA-C*05:01 epitope) [31]	C:3056894_10
	rs27044 exon 15	C > G, E730Q	antigen presentation (binding affinity) [18, 32]	C:3056870_10
	rs30187 exon 11	C > T, R528K	enzymatic activity and selectivity, cellular antigen presentation [18]	C:3056885_10
	rs26653 exon 2	G > C, P127R	enzymatic activity, antigen presentation (alteration in the ligand’s entry pocket) [32]	C:794818_30
	rs7063 intron 19	A > T, intron	enzymatic activity (protein expression) [33]	C:3282786_10
ERAP2 5q15	rs2248374 intron 10	G > A, intron	truncated protein devoid of enzymatic activity [34, 35]	C:25649529_10

### 2.3 Serum concentration of ERAP2

6 ml of venous blood was inserted into BD Vacutainer tubes with a clot activator (Becton Dickinson). After 30 minutes of clotting in RT, samples were centrifuged (1500 RPM for 10 minutes in RT), aliquoted and stored at -70°C for further analysis. Serum ERAP2 concentrations in 79 adult patients and 80 controls were measured by an enzyme-linked immunosorbent assay (ELISA) technique using the Human Endoplasmic Reticulum Aminopeptidase 2 test (E15293h, Wuhan EIAab Science Co, China), according to the manufacturer’s instructions. Detection range and sensitivity of the test were 0.312-20 ng/mL and <0.16 ng/mL, respectively. ERAP2 levels were measured in 100 µl of undiluted serum samples at 450 nm wavelength using the Infinite F50 microplate reader (Tecan Trading AG, Switzerland).

### 2.4 Statistical analysis

Differences in allele and genotype distribution between RA patients and the control group were tested using the two-tailed Fisher’s exact test. For multiple comparisons, the Bonferroni correction was applied (as 0.05/number of comparisons). The odds ratio (OR) and its 95% confidence interval (95% CI) were computed as the measure of effect size. Hardy-Weinberg equilibrium was estimated using the chi-square test with 1 degree of freedom. For HWE analysis, the significance threshold p < 0.05 was adjusted additionally. Haplotype analysis was performed using PLINK software version 1.07, and linkage disequilibrium (LD) was performed using Haploview software version 4.2. Power calculations were obtained using Quanto, version 1.5, with the following options: an unmatched case-control study design: hypothesis, gene only; model of inheritance, log-additive; allele frequencies, rs30187 36.92%, rs27044 31.22%, rs26653 24.68%, rs26618 32.28%, rs7063 26.58%, rs2248374 49.58%; and significance level, 0.05. Differences in serum ERAP2 concentrations between RA patients (or subgroups of patients) and controls are shown in **Supplementary Table 1**. The D’Agostino-Pearson K2 normality test was used to determine if the data distribution deviates from a Gaussian distribution. If the data were normally distributed, the unpaired t-test was used, and if not, the nonparametric Mann-Whitney test was utilized. The required sample size for the Mann-Whitney test was calculated using G Power software version 3.1.9.7 with the following conditions: α error, 0.05; the power of the test (1-β err prob), 80%; effect size, 0.5.

Receiver operating characteristic (ROC) analysis was applied to assess whether tested ERAP2 levels could predict the severity of rheumatoid arthritis. As part of the ROC analysis, the area under the curve (AUC), and the threshold value (T) with optimal sensitivity and specificity of the test were also determined. A p-value < 0.05 was considered significant. All aforementioned statistical analyses were performed in GraphPad Prism ver. 8.1 software (San Diego, CA, USA).

## 3 Results

### 3.1 Distribution of *ERAP1* and *ERAP2* polymorphisms in patients and controls

This study analyzed *ERAP1* and *ERAP2* gene polymorphisms in RA patients and the control group. **Table 3** summarizes the frequency of *ERAP1* and *ERAP2* genotypes in the control group and patients with RA. When comparing the group of patients with RA with the control group, a significant difference was observed in the rs30187 C > T polymorphism of the *ERAP1* gene. The TT genotype was less common in patients with RA than in the control group [8.14% vs. 14.35%, p = 0.025, OR = 0.529, 95% CI (0.304-0.920)], which indicates a protective role of this genotype against RA.

**Table 3:** *ERAP1* and *ERAP2* genotypes and minor allele frequencies in RA patients and control groups.

	RA patients	Control	RA female	Control female	RA male	Control male
**ERAP1 rs30187**	N = 295	N = 237	N = 192	N = 125	N = 103	N = 112
CC	118 (40.00)	96 (40.50)	77 (40.10)	49 (39.20)	41 (39.80)	47 (41.96)
CT	153 (51.86)	107 (45.15)	97 (50.52)	57 (45.60)	56 (54.37)	50 (44.64)
TT	24 (8.14)^1^	34 (14.35)	18 (9.38)	19 (15.20)	6 (5.83)	15 (13.40)
Minor allele T	201 (34.07)	175 (36.92)	133 (34.64)	95 (38.00)	68 (33.01)	80 (35.71)
H-W	p = 0.008	p = 0.636				
**ERAP1 rs27044**	N = 295	N = 237	N = 192	N = 125	N = 103	N = 112
CC	153 (51.86)	110 (46.41)	100 (52.08)	60 (48.00)	53 (51.46)	50 (44.64)
CG	131 (44.41)	106 (44.73)	85 (44.27)	53 (42.40)	46 (44.66)	53 (47.32)
GG	11 (3.73)^2^	21 (8.86)	7 (3.65)^5^	12 (9.60)	4 (3.88)	9 (8.04)
Minor allele G	153 (25.93)	148 (31.22)	99 (25.78)	77 (30.80)	54 (26.21)	71 (31.70)
H-W	p = 0.007	p = 0.524				
**ERAP1 rs26653**	N = 295	N = 237	N = 192	N = 125	N = 103	N = 112
GG	154 (52.21)	139 (58.65)	94 (48.96)	71 (56.80)	60 (58.25)	68 (60.71)
CG	116 (39.32)	79 (33.33)	79 (41.14)	43 (34.40)	37 (35.92)	36 (32.15)
CC	25 (8.47)	19 (8.02)	19 (9.90)	11 (8.80)	6 (5.83)	8 (7.14)
Minor allele C	166 (28.14)	117 (24.68)	117 (30.47)	65 (26.00)	49 (23.79)	52 (23.21)
H-W	p = 0.635	p = 0.111				
**ERAP1 rs26618**	N = 295	N = 237	N = 192	N = 125	N = 103	N = 112
TT	157 (53.22)^3^	104 (43.88)	107 (55.73)^6^	49 (39.20)	50 (48.54)	55 (49.11)
CT	113 (38.31)^4^	113 (47.68)	70 (36.46)^7^	62 (49.60)	43 (41.75)	51 (45.54)
CC	25 (8.47)	20 (8.44)	15 (7.81)	14 (11.20)	10 (9.71)	6 (5.35)
Minor allele C	163 (27.63)	153 (32.28)	100 (26.04)	90 (36.00)	63 (30.58)	63 (28.13)
H-W	p = 0.470	p = 0.163				
**ERAP1 rs7063**	N = 295	N = 237	N = 192	N = 125	N = 103	N = 112
AA	148 (50.17)	124 (52.32)	103 (53.65)	72 (57.60)	45 (43.69)	52 (46.43)
AT	128 (43.39)	100 (42.19)	78 (40.62)	46 (36.80)	50 (48.54)	54 (48.21)
TT	19 (6.44)	13 (5.49)	11 (5.73)	7 (5.60)	8 (7.77)	6 (5.36)
Minor allele T	166 (28.14)	126 (26.58)	100 (26.04)	60 (24.00)	66 (32.04)	66 (29.46)
H-W	p = 0.210	p = 0.212				
**ERAP2 rs2248374**	N = 295	N = 237	N = 192	N = 125	N = 103	N = 112
AA	62 (21.01)	64 (27.00)	38 (19.79)^8^	40 (32.00)	24 (23.30)	24 (21.42)
AG	160 (54.24)	111 (46.84)	102 (53.13)	53 (42.40)	58 (56.31)	58 (51.79)
GG	73 (24.75)	62 (26.16)	52 (27.08)	32 (25.60)	21 (20.39)	30 (26.79)
Minor allele G	306 (51.86)	235 (49.58)	206 (53.65)	117 (46.80)	106 (48.54)	118 (52.68)
H-W	p = 0.138	p = 0.330				

H-W, Hardy-Weinberg equilibrium; p, probability; OR, odds ratio; 95% CI, confidence interval from two-sided Fisher’s exact test. The statistically significant threshold was set at p < 0.0028 after Bonferroni correction (0.05/18 comparisons). Values in parentheses are in percentages.

RA vs. Control: ^1^p = 0.025, OR = 0.529, 95% CI (0.304-0.920); ^2^p = 0.017, OR = 0.398, 95% CI (0.188-0.844); ^3^p = 0.036, OR = 1.455, 95% CI (1.032-2.052); ^4^p = 0.034, OR = 0.681, 95% CI (0.482-0.964); ^5^p = 0.049, OR = 0.356, 95% CI (0.136-0.932); ^6^p = 0.004, OR = 1.952, 95% CI (1.235-3.088); ^7^p = 0.027, OR = 0.583, 95% CI (0.369-0.921); ^8^p = 0.016, OR = 0.524, 95% CI (0.313-0.879).

Significant differences were also observed in the rs27044 C > G polymorphic site of the *ERAP1* gene for the GG genotype, which was less frequent in the group of patients with RA compared to controls [3.73% vs. 8.86%, p = 0.017, OR = 0.398, 95% CI (0.188-0.844)].

RA patients also differed in the rs26618 *ERAP1* TT polymorphism [53.22% vs. 43.88%, p = 0.036, OR = 1.455, 95% CI (1.032-2.052)] and CT [38.31% vs. 47.68%, p = 0.034, OR = 0.681, 95% CI (0.482-0.964)]. The results showed that the TT genotype predisposes to RA, while the CT genotype protects against this disease.

For the remaining tested polymorphisms, we did not find any differences between the RA patient group and controls.

It is known that women suffer from RA more frequently than men, so we decided to analyze the studied polymorphisms in terms of gender. We found significant differences for the GG rs27044 *ERAP1* genotype, and a lower frequency of this genotype was observed in women with RA compared to control women [3.65% vs. 9.60%, p = 0.049, OR = 0.356, 95% CI (0.136-0.932)]. Moreover, an increased frequency of the TT genotype in rs26618 was found in female patients [55.73% vs. 39.20%, p = 0.004, OR = 1.952, 95% CI (1.235-3.088)] alongside a reduced frequency of the CT genotype [36.46% vs. 49.60%, p = 0.027, OR = 0.583, 95% CI (0.369-0.921)] compared to control women.

Women with RA also differed from healthy women in the *ERAP2* rs2248374 G > A polymorphism. A lower frequency of the AA genotype was noticed in women with RA than in control women [19.79% vs. 32.00%, p = 0.016, OR = 0.524, 95% CI (0.313-0.879)].

However, all observed differences in **Table 3** lost significance after Bonferroni correction for multiple comparisons. No significant differences were found in the *ERAP1* and *ERAP2* gene polymorphisms in men with RA and men in the control group.

The analysis of RA patients according to the severity of the disease and DAS28 (Disease Activity Score 28) showed differences between patient subgroups in the *ERAP1* rs26653 G > C SNP (p = 0.043/p_corr._= ns) and *ERAP2* rs2248374 G > A (p = 0.003) (**Table 4**). The rs26653 GG genotype was more common in patients in remission or a mild disease phase (DAS28 < 3.2 – 66.67%), while the CG genotype was the most frequent in patients with moderate disease (DAS28 = 3.2-5.1 – 48.15%). In the case of *ERAP2* rs2248374 G > A, patients with the AA genotype significantly increased in frequency from mild (DAS28 < 3.2) through moderate (DAS28 = 3.2-5.1) to severe disease (DAS28 > 5.1) (10.42%, 19.26%, and 27.68%, respectively).

**Table 4:** *ERAP1* and *ERAP2* genotypes in patients according to Disease Activity Score 28 (DAS28).

	DAS 28 < 3.2	DAS 28 3.2-5.1	DAS 28 > 5.1	p	χ^2^
**ERAP1 rs30187**	N = 48	N = 135	N = 112		
CC	16 (33.33)	55 (40.74)	47 (41.96)		
CT	28 (58.33)	70 (51.85)	55 (49.11)	p = 0.846	
TT	4 (8.34)	10 (7.41)	10 (8.93)		
**ERAP1 rs27044**	N = 48	N = 135	N = 112		
CC	21 (43.75)	77 (57.04)	55 (49.11)		
CG	24 (50.00)	53 (39.26)	54 (48.21)	p = 0.389	
GG	3 (6.25)	5 (3.70)	3 (2.68)		
**ERAP1 rs26653**	N = 48	N = 135	N = 112		
CC	4 (8.33)	10 (7.41)	11 (9.82)		
CG	12 (25.00)	65 (48.15)	39 (34.82)	p = 0.043	9.871
GG	32 (66.67)	60 (44.44)	62 (55.36)		
**ERAP1 rs26618**	N = 48	N = 135	N = 112		
CC	2 (4.17)	11 (8.15)	12 (10.71)		
CT	20 (41.67)	48 (35.55)	45 (40.18)	p = 0.584	
TT	26 (54.16)	76 (56.30)	55 (49.11)		
**ERAP1 rs7063**	N = 48	N = 135	N = 112		
AA	25 (52.08)	68 (50.37)	55 (49.11)		
AT	22 (45.83)	57 (42.22)	49 (43.75)	p = 0.760	
TT	1 (2.09)	10 (7.41)	8 (7.14)		
**ERAP2 rs2248374**	N = 48	N = 135	N = 112		
AA	5 (10.42)	26 (19.26)	31 (27.68)		
AG	33 (68.75)	64 (47.41)	63 (56.25)	**p = 0.003**	**15.964**
GG	10 (20.83)	45 (33.33)	18 (16.07)		

p – probability and χ2 were estimated from the Chi-squared Test for Independence. The statistically significant threshold was set at p < 0.008 after Bonferroni correction (0.05/6 comparisons). The value in bold indicates a significant difference. Values in parentheses are in percentages.

The next step was to analyze the distribution of the tested polymorphisms in patients with the presence or absence of RA diagnostic markers such as RF, anti-cyclic citrullinated peptide antibodies (anti-CCP), and anti-mutated citrullinated vimentin antibodies (anti-MCV) (**Table 5**). We observed a difference for the GG rs2248374 ERAP2 in RF-positive patients compared to RF-negative [29.80% vs. 19.44%, p = 0.044, OR = 1.759, 95% CI (1.024-3.019)]. We also detected weak differences in anti-CCP-positive patients compared to anti-CCP-negative for *ERAP1* rs26653 GG [47.13% vs. 59.50%, p = 0.044, OR = 0.607, 95% CI (0.379-0.970)] and CG [44.25% vs. 32.23%, p = 0.040, OR = 1.669, 95% CI (1.028-2.710]), respectively. These comparisons were not significant after the Bonferroni correction.

**Table 5:** *ERAP1* and *ERAP2* genotypes in RA patients with different diagnostic markers (RF, anti-CCP, and anti-MCV).

	RF-positive	RF-negative	anti-CCP-positive	anti-CCP-negative	anti-MCV-positive	anti-MCV-negative
**ERAP1 rs30187**	N = 151	N = 144	N = 174	N = 121	N = 151	N = 144
CC	64 (42.38)	54 (37.50)	72 (41.38)	46 (38.02)	52 (34.44)	66 (45.83)
CT	75 (49.67)	78 (54.17)	88 (50.57)	65 (53.72)	87 (57.62)^4^	66 (45.83)
TT	12 (7.95)	12 (8.33)	14 (8.05)	10 (8.26)	12 (7.94)	12 (8.34)
**ERAP1 rs27044**	N = 151	N = 144	N = 174	N = 121	N = 151	N = 144
CC	84 (55.63)	69 (47.92)	98 (56.32)	55 (45.46)	77 (50.99)	76 (52.78)
CG	62 (41.06)	69 (47.92)	70 (40.23)	61 (50.41)	69 (45.70)	62 (43.06)
GG	5 (3.31)	6 (4.16)	6 (3.45)	5 (4.13)	5 (3.31)	6 (4.16)
**ERAP1 rs26653**	N = 151	N = 144	N = 174	N = 121	N = 151	N = 144
CC	13 (8.61)	12 (8.33)	15 (8.62)	10 (8.27)	13 (8.61)	12 (8.33)
CG	67 (44.37)	49 (34.03)	77 (44.25)^2^	39 (32.23)	**74 (49.01)^5^**	42 (29.17)
GG	71 (47.02)	83 (57.64)	82 (47.13)^3^	72 (59.50)	**64 (42.38)^6^**	90 (62.50)
**ERAP1 rs26618**	N = 151	N = 144	N = 174	N = 121	N = 151	N = 144
CC	13 (8.61)	12 (8.33)	13 (7.47)	12 (9.92)	9 (5.96)	16 (11.11)
CT	51 (33.77)	62 (43.06)	63 (36.21)	50 (41.32)	62 (41.06)	51 (35.42)
TT	87 (57.62)	70 (48.61)	98 (56.32)	59 (48.76)	80 (52.98)	77 (53.47)
**ERAP1 rs7063**	N = 151	N = 144	N = 174	N = 121	N = 151	N = 144
AA	76 (50.33)	72 (50.00)	87 (50.00)	61 (50.41)	80 (52.98)	68 (47.22)
AT	65 (43.04)	63 (43.75)	77 (44.25)	51 (42.15)	68 (45.03)	60 (41.67)
TT	10 (6.62)	9 (6.25)	10 (5.75)	9 (7.44)	**3 (1.99)^7^**	16 (11.11)
**ERAP2 rs2248374**	N = 151	N = 144	N = 174	N = 121	N = 151	N = 144
AA	26 (17.22)	36 (25.00)	35 (20.11)	27 (22.31)	30 (19.87)	32 (22.22)
AG	80 (52.98)	80 (55.56)	92 (52.87)	68 (56.20)	84 (55.63)	76 (52.78)
GG	45 (29.80)^1^	28 (19.44)	47 (27.02)	26 (21.49)	37 (24.50)	36 (25.00)

p, probability; OR, odds ratio; 95% CI, confidence interval from two-sides Fisher’s exact test. The statistically significant threshold was set at p < 0.0028 after Bonferroni correction (0.05/18 comparisons). Value in bold indicates significant differences. Values in parentheses are in percentages.

RF-positive vs. RF-negative: ^1^p = 0.044, OR = 1.759, 95% CI (1.024-3.019);

Anti-CCP-positive vs. anti-CCP-negative: ^2^p = 0.040, OR = 1.669, 95% CI (1.028-2.710); ^3^p = 0.044, OR = 0.607, 95% CI (0.379-0.970);

Anti-MCV-positive vs. anti-MCV-negative: ^4^p = 0.048, OR = 1.607, 95% CI (1.014-2.545); ^5^p = 0.0005, OR = 2.334, 95% CI (1.443-3.774); ^6^p = 0.0007, OR = 0.441, 95% CI (0.277-0.704); ^7^p = 0.0015, OR = 0.162, 95% CI (0.046-0.569).

In the case of rs30187 *ERAP1* CT, we noted a higher percentage of anti-MCV-positive patients than anti-MCV-negative [57.62% vs. 45.83%, p = 0.048/pcorr. = ns, OR = 1.607, 95% CI (1.014-2.545)]. Additionally, in the rs26653 polymorphism, the GG genotype was significantly less common in anti-MCV-positive patients [42.38% vs. 62.50%, p = 0.0007, OR = 0.441, 95% CI (0.277-0.704)] and the CG genotype was more widespread in those patients [49.01% vs. 29.17%, p = 0.0005, OR = 2.334, 95% CI (1.443-3.774)]. In turn, in the rs7063 A > T polymorphism, the frequency of the TT genotype was higher in anti-MCV-negative patients than in anti-positive patients [11.11% vs. 1.99%, p = 0.0015, OR = 0.162, 95% CI (0.046-0.569)]. These comparisons retained their significance after applying the correction for the multiple comparisons.

We also performed haplotypes analysis which did not show a significant association of haplotypes with RA (**Supplementary Table 2**; Omnibus p-value for all haplotypes = 0.113). Moreover, **Supplementary Figure 1** shows the linkage disequilibrium (LD) pattern of the six studied SNPs in the *ERAP1* and *ERAP2* genes in patients and controls.

### 3.2 ERAP2 secretion in patients and controls

**Figure 1:**
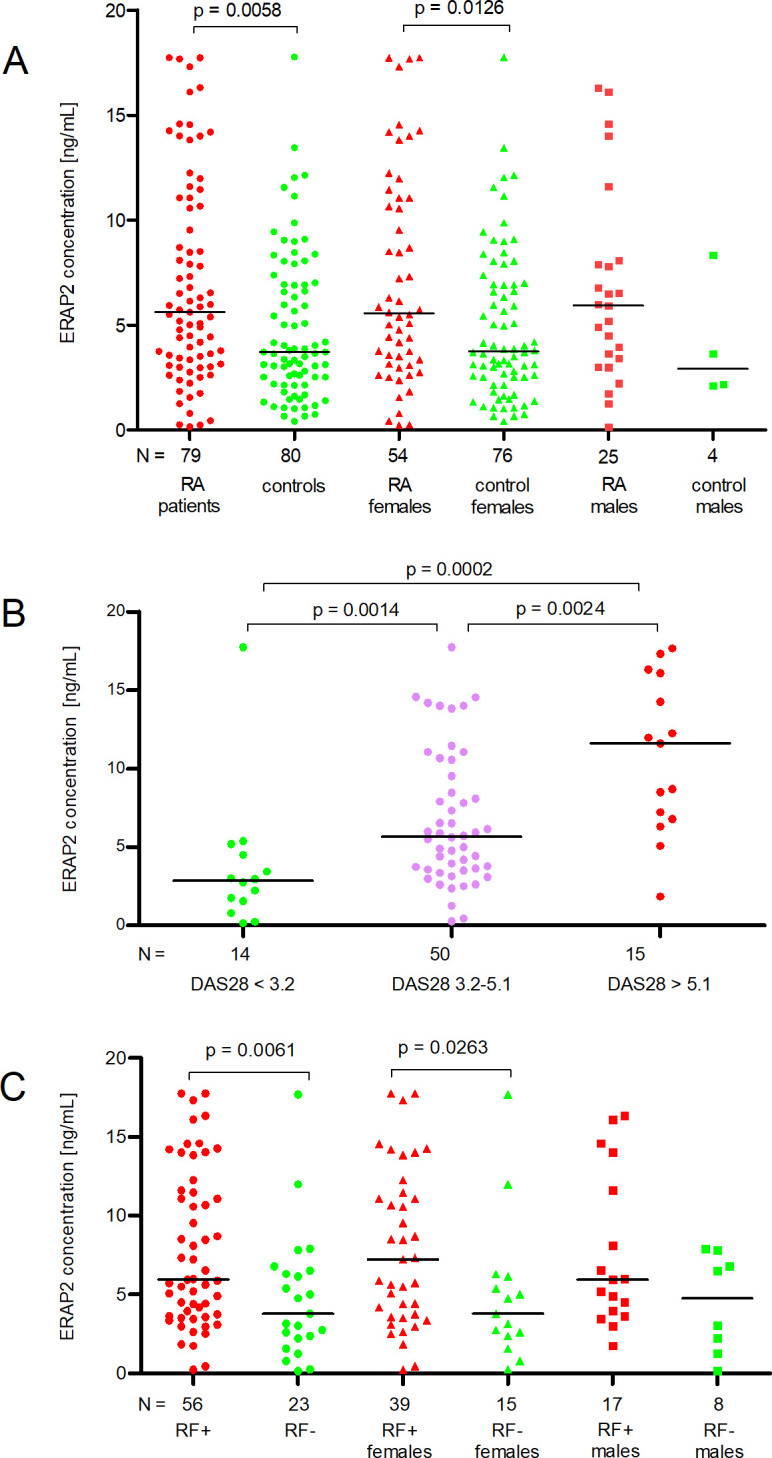
ERAP2 concentration (ng/mL) in patients and controls according to gender **(A)**, degree of disease activity DAS28 **(B)**, and presence of rheumatoid factor (RF) **(C)**. N is the number of patients. Black horizontal lines represent medians. P-values are calculated by the Mann-Whitney test (except for DAS28 3.2-5.1 vs. DAS > 5.1 comparison, it uses an unpaired t-test). Red points mean measurement in RA patients or patients with DAS28 > 5.1, or RF-positive patients; green points mean measurement in controls or patients with DAS28 < 3.2, or RF-negative patients; purple points mean measurement in patients with DAS28 = 3.2-5.1.

We found that RA patients secreted higher concentrations of ERAP2 than controls (Median 5.61 vs. 3.71 ng/mL). The difference was statistically significant (p = 0.0058, **Figure 1A**). We also detected a significantly increased level of ERAP2 in the serum of RA females compared to control females (Median 5.56 vs. 3.76 ng/mL, p = 0.0126, **Figure 1A**).

Although the median ERAP2 concentrations in males with RA and males in the control group differed twofold, we did not note a significant difference (5.93 vs. 2.92 ng/ml, **Figure 1A**) due to too few males in the control group (N = 4).

When we divided patients according to DAS28, we observed a linear increase in ERAP2 levels in patients with DAS < 3.2 through 3.2-5.1 and finally > 5.1 (Medians 2.86-5.67-11.59 ng/mL, respectively). The comparison of DAS28 < 3.2 patients with DAS28 3.2-5.1 and DAS28 3.2-5.1 with DAS28 > 5.1 was meaningful (p = 0.0014, and p = 0.0024, respectively, **Figure 1B**). It should be underlined that patients with mild activity (DAS < 3.2) secreted a concentration of ERAP2 four times lower than in patients with severe disease (DAS28 > 5.1) (p = 0.0002, **Figure 1B**).

Finally, the last analysis concerns patients divided according to RF. We detected a higher level of ERAP2 in RF-positive patients than in RF-negative patients (Medians 5.97 vs. 3.77, p = 0.0061). Also, RF-positive females secreted higher concentrations than RF-negative (Medians 7.22 vs. 3.77 ng/mL, p = 0.0263, **Figure 1C**).

### 3.3 Receiver operating characteristic

**Figure 2:**
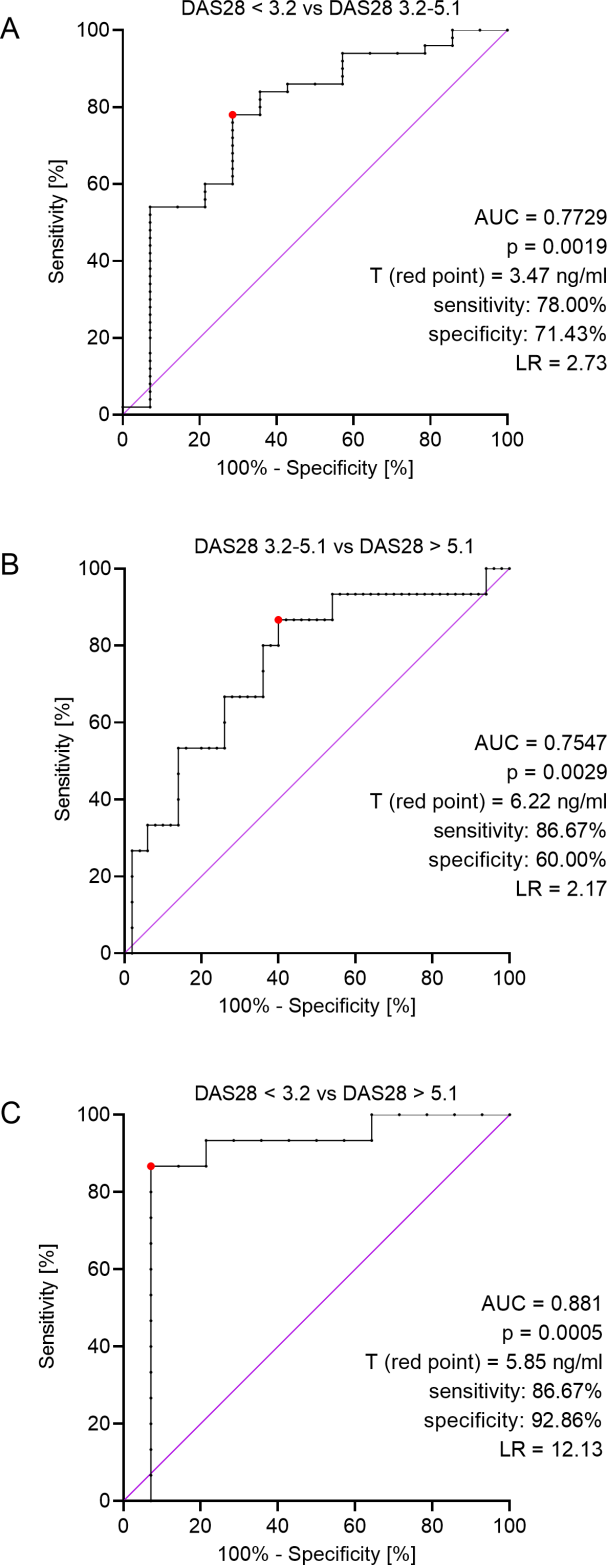
ROC analyses for: patients in remission or a mild phase of the disease (DAS28 < 3.2) and patients with moderate RA (DAS28 = 3.2-5.1) **(A)**, patients with moderate (DAS28 = 3.2-5.1) and severe (DAS28 > 5.1) stage of the disease **(B)**, patients in remission or a mild phase of the disease (DAS28 < 3.2) and patients with severe phase of the disease (DAS28 > 5.1) **(C)**, AUC – area under curve, T (red point) – threshold value, LR – likelihood ratio.

We performed ROC analysis for patients in all stages of the disease to find a concentration of ERAP2 that would differentiate these three stages. First, we compared those patients with remission/mild (DAS 28 < 3.2) and middle (DAS 28 = 3.2-5.1) phases of the disease and found that a concentration above 3.47 ng/mL indicates middle phase RA (area under the curve (AUC) = 0.7729, 95% CI 0.63-0.92, p = 0.0019, likelihood ratio (LR) = 2.73; **Figure 2A**). Then, we analyzed patients with middle (DAS 3.2-5.1) and severe (DAS > 5.1) stages of the disease and revealed that a concentration above 6.22 ng/mL indicates a severe phase of RA (AUC = 0.7547, 95% CI 0.62-0.90, p = 0.0029, LR = 2.17; **Figure 2B**). Finally, we compared patients with remission/mild (DAS < 3.2) and severe (DAS > 5.1) stages of the disease and revealed that a concentration above 5.85 ng/mL indicates severe RA (AUC = 0.8810, 95% CI 0.73-1.00, p = 0.0005, LR = 12.13; **Figure 2C**). This test seems to be the most valuable in terms of diagnostics due to its higher AUC and LR reliability index.

## 4 Discussion

It’s known that RA is an inflammatory multi-factorial autoimmune disease that mainly harms small joints and progresses into erosive-destructive polyarthritis. The disease evolves in middle and older ages, primarily in women, and is characterized by painful and autoinflammatory status with prolonged disorders. Several genes are known, and some polymorphisms are associated with progression of RA (5, 36, 37). The ERAP1 and ERAP2 genes encode endoplasmic reticulum aminopeptidases, forming peptides that create antigens presented by HLA class I molecules. The polymorphism of these genes influences the diverse functions of aminopeptidases and affects the formation of HLA-antigen complexes (28, 33).

The human *ERAP1* and *ERAP2* genes are encoded in the short arm chromosome 5q15 in the opposite orientation, and they likely have two shared regulatory components. *ERAP1* is highly polymorphic, with strong linkage disequilibrium apparent across the gene. Some of the single nucleotide polymorphisms (SNPs) present in *ERAP1* may cause changes in the gene expression and alter the enzymatic activity and substrate binding (29, 38). The potential impact of some of these variants on ERAP1 biological functions can be inferred from their location in the protein structure. For example, rs27044 (Q730E) is exposed on the inner area of the C-terminal cavity, which could affect substrate sequence or length specificity. Other polymorphisms, such as rs26653 (R127P) and rs30187 (K528R) (alteration in the ligand’s entry pocket), could indirectly affect either substrate specificity or enzymatic activity by changing the conformational motives between the open and closed form of the enzyme (17). Rs7063 which is present in the middle of a conserved transcription termination sequence (AATAAA) in the 3`-UTR of *ERAP1* 19E isoform, may be involved in the transcription termination process (33). Finally, the rs26618 (Ile276Met) may affect ERAP1 enzymatic activity. Combinations of SNPs in the *ERAP1* gene form 10 haplotypes of different activity (39, 40).

A significant functional difference in the rs30187 C/T (R528K) polymorphism of the *ERAP1* gene diminishes the efficacy of peptide trimming by changing ERAP1 from an active to an inactive form (18). Here, the TT genotype was less common in Ukrainian patients with RA than in the control group, indicating this genotype’s protective role against RA. On the contrary, in Akbulut et al. (2022), the TT genotype was associated with RA disease risk in the Turkish population (32). The association of the rs30187 polymorphism with susceptibility to different diseases other than RA has been shown in many studies. An elevated disease risk for TT genotype-positive patients was observed in ankylosing spondylitis (AS) in the Polish population by Wisniewski et al. (41) and also in several other autoimmune diseases in different populations, Caucasian or Han Chinese (42).

In the case of *ERAP1* rs27044 C > G (Q730E), we found the same pattern as in the case of rs30187 – the GG genotype was seen less frequently in patients with RA than in the control group, indicating the protective role of this genotype against RA. Rs27044 polymorphism is also significantly associated with an increased risk of AS, especially in some Caucasians, Han Chinese, and Koreans (43, 44), and spondyloarthritis in the French population, but not in the Belgian population (42).

We also found some other significant results concerning the *ERAP1* gene: RA patients homozygotic for rs26618T were more often observed than in the control group, indicating an association with predisposition to the disease. The opposite was observed with the CT genotype demonstrating a protective effect. For cervical cancer, the CC genotype of rs26618 was a risk factor compared with the TT or CT genotype in the Chinese population (45) but not in the Dutch (46). We previously found an association of the rs26618*C allele and the rs26618*CC genotype with atopic dermatitis in the Polish population. In addition, as rs26618*C encodes the 276Met variant, we found that the enzyme with this substitution produces a peptide for the HLA-C*15 molecule, also associated with atopic dermatitis in the Polish population, with two times lower activity than the major variant, 276Ile (31).

It should be also mentioned that some *ERAP1* polymorphisms tested in our study were associated with disease activity and other clinical manifestations such as the presence of anti-CCP and anti-MCV antibodies. We found the rs26653 GG genotype more frequent in patients with a mild stage of disease (DAS28 < 3.2) in comparison to more severe stages of disease. In addition, this genotype was more frequent in anti-CCP negative and anti-MCV negative patients. This indicates protection against disease progression.

We must underline that in our study, the distribution of all tested SNPs in controls was consistent with the HWE, indicating correct genotyping. According to the National Library of Medicine, the frequency of the T rs30187 minor allele in the Ukrainian population (36.9%) was only slightly different from the European population (35.3%) [https://www.ncbi.nlm.nih.gov/snp/rs30187]. The frequency of the G allele in the rs27044 polymorphism in the Ukrainian population was 31%, while it was 27% in other Caucasians.

How can we explain the differences observed in our research and others on the role of *ERAP1* gene in rheumatic diseases, especially RA and AS? HLA class II alleles are generally connected to seropositive diseases: several autoimmune conditions (including coeliac disease, T1D, autoimmune thyroid disease, systemic lupus erythematosus (SLE), and others) are related to the HLA-DR3-DQ2 haplotype. RA is associated with different alleles of HLA-DRB1. By contrast, seronegative diseases are generally correlated with HLA class I alleles, which are disease-specific (i.e., AS is associated with HLA-B27 (47, 48) and psoriasis with HLA-Cw6 (49), etc.). In our study, the percentage of seropositive and negative patients was almost equal. It suggests that both pathomechanisms might be possible.

In contrast to *ERAP1*, *ERAP2* shows limited polymorphism. The two major *ERAP2* isoforms result from an alternative splicing in correspondence to the pivotal SNP rs2248374 (A/G) present within the 5′-splice site of exon 10. The *ERAP2-A* isoform (A allele) encodes a full-length and functional protein, whereas the *ERAP2-B* isoform (G allele) is degraded by nonsense-mediated decay (50, 51). Since both alleles of rs2248374 have similar frequency in most populations, only three-fourths of individuals [AA homozygotes (25%) and AG heterozygotes (50%)] express a functional ERAP2 isoform, while the remaining individuals (with GG genotype – occurring in ~25% of the population) express two undetectable isoforms of ERAP2-B, resulting in reduced MHC class I surface expression in lymphoblastoid cell lines (50). In addition, viral and also perhaps bacterial infections induce, in rs2248374G individuals, the expression of a third truncated transcript giving a shortened protein devoid of enzymatic activity but nevertheless possibly capable of interfering with ERAP1 and ERAP2 function (14, 34, 35). The polymorphism of the *ERAP2* gene was associated with several immune-mediated diseases including AS (41, 52), psoriasis (21, 22) and preeclampsia (53). Interestingly, as *ERAP1* and *ERAP2* genes are closely linked on chromosome 5q15, the defective *ERAP2* allele is frequently correlated to a highly active *ERAP1* haplotype, and vice versa (40).

During our study, we observed a protective role of the *ERAP2* rs2248374 AA genotype in RA susceptibility, but it was only seen in women. However, when analyzing the distribution of *ERAP2* rs2248374 genotypes depending on disease activity, we found that the frequency of the AA genotype was highest in patients with severe disease compared to patients with mild disease. This rather points to the *ERAP2 A* haplotype and the production of full-length ERAP2 proteins as a risk for RA progression. This observation is consistent with other studies showing that the *ERAP2 A* haplotype increases the risk of autoimmune diseases such as Crohn’s disease, juvenile idiopathic arthritis (JIA), and BSCR (54). Additionally, in our study, RF-positive patients exhibited a significantly higher frequency of the GG genotype (no active ERAP2) than RF-negative which suggests that the lack of the functional enzyme may favor the production of RF. Thus, we may speculate that ERAP2 (encoded by rs2248374A allele) eliminates rheumatogenic peptide(s) including those stimulating RF production, but once the patient gets RA, then ERAP2 may produce peptides aggravating the disease or destroy protective peptides.

Moreover, we found that RA patients secreted higher concentrations of ERAP2 than controls. We also observed a linear increase in ERAP2 levels in patients according to DAS28, suggesting a role of the ERAP2 protein in the severity of RA. Additionally, ROC analysis indicated 5.85 ng/mL of ERAP2 as a point of severe RA development. It is known that pro-inflammatory cytokines such as IFNs and TNF-α significantly increase the expression of ERAP2 (55, 56). Chronic inflammation in RA supports increased expression and secretion of ERAP2, as observed in our study. Furthermore, additional SNPs influence ERAP2 expression, altering enhancer-promoter interactions and affecting expression (57). These SNPs deserve further study.

However, it should be emphasized that our study possesses some limitations. First, the number of patients and controls is too small for SNP analysis. Power calculations showed that to achieve 80% power, it would be necessary to test 2357 patients and 1892 controls for rs30187, 634 patients and 509 controls for rs27044, 1506 patients and 1209 controls for rs26653, 845 patients and 678 controls for rs26618, 7498 patients and 6020 controls for rs7063, and 3883 patients and 3118 controls for rs2248374. In turn, power test analysis for ERAP2 concentration showed that to achieve a result of 80% we needed 75 subjects in each group, which was met in our study. Moreover, a potential confounding factor in the study may be the fact that the control group was not fully matched in terms of age (p < 0.0002 in comparison to the patients) and sex (p < 0.0045 in comparison to the patients). However, age does not influence SNP genotype. Also, results in ERAP2 secretion were independent of sex. RA females and males possessed similar concentrations of ERAP2 (**Figure 1A**). The factors determining the secretion of ERAP2 were DAS28 (**Figure 1B**) and RF (**Figure 1C**).

In conclusion, we found genetic differences in *ERAP1* and *ERAP2* genes between RA patients and healthy controls. Additionally, the ERAP2 protein tested in serum could be a valuable biomarker of RA severity.

## Data Availability

The raw data supporting the conclusions of this article will be made available by the authors, without undue reservation.
