# Supplementary material for: Association of ERAP1 and ERAP2 gene polymorphisms and ERAP2 protein with the susceptibility and severity of rheumatoid arthritis in the Ukrainian population
Source: Front Immunol. 2025 Jan 21;15:1519159. doi: 10.3389/fimmu.2024.1519159 (PMC11790443; doi:10.3389/fimmu.2024.1519159)
Supplement: Supplementary file 1 [file Table1.docx]

**Supplementary Table 1** ERAP2 secretion in RA patients and controls, including RA females and males, RF-positive and negative, and patients with varying DAS28 activity

| **Study groups** | **RA patients** | **Control** | **RA female** | **Control female** | **RA male** | **Control male** | **DAS28 < 3.2** | **DAS28 3.2-5.1** | **DAS28 > 5.1** | **RF+** | **RF-** | **RF+ female** | **RF-female** | **RF+ male** | **RF-male** |
| --- | --- | --- | --- | --- | --- | --- | --- | --- | --- | --- | --- | --- | --- | --- | --- |
| Number of patients | 79 | 80 | 54 | 76 | 25 | 4 | 14 | 50 | 15 | 56 | 23 | 39 | 15 | 17 | 8 |
| Minimum | 0.15 | 0.41 | 0.23 | 0.41 | 0.15 | 2.12 | 0.15 | 0.26 | 1.84 | 0.23 | 0.15 | 0.23 | 0.26 | 1.75 | 0.15 |
| 25% Percentile | 3.14 | 2.27 | 3.13 | 2.53 | 3.22 | 2.14 | 1.37 | 3.55 | 6.78 | 3.66 | 2.23 | 3.56 | 2.37 | 3.79 | 1.50 |
| Median | 5.61 | **3.71^a^** | 5.56 | **3.76^b^** | 5.93 | 2.92 | 2.86 | **5.67^c^** | **11.59^d,e^** | 5.97 | **3.77^f^** | 7.22 | **3.77^g^** | 5.93 | 4.76 |
| 75% Percentile | 10.70 | 6.94 | 11.10 | 6.94 | 7.99 | 7.16 | 4.67 | 9.78 | 16.10 | 11.60 | 6.50 | 11.50 | 6.14 | 12.80 | 7.55 |
| Maximum | 17.70 | 17.80 | 17.70 | 17.8 | 16.3 | 8.33 | 17.70 | 17.70 | 17.70 | 17.70 | 17.70 | 17.70 | 17.7 | 16.30 | 7.90 |
| Mean | 6.94 | 4.83 | 7.10 | 4.87 | 6.60 | 4.07 | 3.69 | 6.70 | 10.81 | 7.83 | 4.79 | 7.92 | 4.97 | 7.61 | 4.45 |
| Std. Deviation | 4.86 | 3.48 | 5.01 | 3.51 | 4.60 | 2.93 | 4.37 | 4.25 | 4.90 | 4.92 | 4.03 | 4.99 | 4.53 | 4.91 | 3.13 |
| Std. Error of Mean | 0.55 | 0.39 | 0.68 | 0.40 | 0.92 | 1.46 | 1.17 | 0.60 | 1.26 | 0.66 | 0.84 | 0.80 | 1.17 | 1.19 | 1.11 |
| Lower 95% CI of mean | 5.86 | 4.05 | 5.73 | 4.07 | 4.70 | -0.59 | 1.17 | 5.49 | 8.08 | 6.51 | 3.05 | 6.31 | 2.46 | 5.09 | 1.84 |
| Upper 95% CI of mean | 8.03 | 5.60 | 8.47 | 5.67 | 8.50 | 8.73 | 6.21 | 7.91 | 13.51 | 9.15 | 6.53 | 9.54 | 7.47 | 10.10 | 7.07 |
| D'Agostino & Pearson omnibus normality test K^2^ | 7.95 | 19.32 | 47.73 | 18.23 | 3.99 | N too small | 27.68 | 5.43 | 1.23 | 7.80 | 15.90 | 4.11 | 14.38 | 3.09 | 3.33 |

P-values are calculated by the Mann-Whitney test (except for DAS28 3.2-5.1 vs. DAS > 5.1 comparison, which utilized an unpaired t-test)

**RA patients vs. controls:** ^a^p = 0.0058

**RA female vs. control female:** ^b^p = 0.0126

**DAS28 < 3.2 vs. DAS28 3.2-5.1:** ^c^p = 0.0014

**DAS28 3.2-5.1 vs. DAS28 > 5.1:** ^d^p = 0.0024

**DAS < 3.2 vs. DAS28 > 5.1:** ^e^p = 0.00002

**RF+ patients vs. RF- patients:** ^f^p = 0.0061

**RF+ females vs. RF- females:** ^g^p = 0.0263
