# Supplementary material for: Association of ERAP1 and ERAP2 gene polymorphisms and ERAP2 protein with the susceptibility and severity of rheumatoid arthritis in the Ukrainian population
Source: Front Immunol. 2025 Jan 21;15:1519159. doi: 10.3389/fimmu.2024.1519159 (PMC11790443; doi:10.3389/fimmu.2024.1519159)
Supplement: Supplementary file 2 [file Table2.docx]

**Supplementary Table 2**. Estimated ERAP1/2 haplotype frequencies for RA patients (N = 295) and healthy controls (N = 237)

| **Haplotype ID** | **Haplotype** | **Patients [%]** | **Controls**  **[%]** | **p** | **p_corr._** | **OR** | **95% CI** |
| --- | --- | --- | --- | --- | --- | --- | --- |
| h1 | AGTTCA | 3.48 | 2.28 | 0.361 | ns | 1.48 | 0.70-3.11 |
| h2 | ACTTCA | 6.04 | 3.67 | 0.050 | ns | 1.80 | 1.00-3.24 |
| h3 | ACCTCA | 2.46 | 1.20 | 0.256 | ns | 1.90 | 0.73-4.97 |
| h4 | TCCCGA | 1.57 | 1.90 | 0.642 | ns | 0.80 | 0.32-2.03 |
| h5 | ACCCGA | 18.78 | 22.68 | 0.146 | ns | 0.79 | 0.59-1.07 |
| h6 | AGTTGA | 1.64 | 3.19 | 0.153 | ns | 0.53 | 0.23-1.19 |
| h7 | TCCTGA | 10.95 | 12.42 | 0.502 | ns | 0.87 | 0.60-1.27 |
| h8 | ACCTGA | 4.12 | 5.07 | 0.460 | ns | 0.80 | 0.45-1.42 |
| h9 | AGTTCG | 7.49 | 10.93 | 0.053 | ns | 0.65 | 0.43-1.00 |
| h10 | ACTTCG | 2.50 | 2.13 | 0.689 | ns | 1.21 | 0.54-2.72 |
| h11 | ACCTCG | 6.36 | 3.28 | 0.034 | ns | 1.92 | 1.05-3.49 |
| h12 | ACCCGG | 6.03 | 5.92 | 1.000 | ns | 1.04 | 0.62-1.72 |
| h13 | AGTTGG | 11.40 | 11.51 | 1.000 | ns | 1.00 | 0.68-1.46 |
| h14 | TCCTGG | 15.49 | 12.36 | 0.184 | ns | 1.28 | 0.90-1.83 |
| h15 | ACCTGG | 1.68 | 1.46 | 0.812 | ns | 1.15 | 0.43-3.05 |

*Haplotypes were estimated in the following order: rs7063A/T|rs27044C/G|rs30187C/T|rs26618C/T|rs26653C/G|rs2248374A/G.

Omnibus p-value for all haplotypes = 0.113 (CHISQ = 20.59, df = 14); haplotypes with frequency < 1% in both patients and controls have been dropped; **p** – probability; **p_corr._** – probability after Bonferroni correction for 15 possible haplotypes; **OR** – odds ratio; **95%** **CI** – confidence interval from two-sided Fisher’s exact test; **ns** – not significant.
