# Supplementary material for: Association of ERAP1 and ERAP2 gene polymorphisms and ERAP2 protein with the susceptibility and severity of rheumatoid arthritis in the Ukrainian population
Source: Front Immunol. 2025 Jan 21;15:1519159. doi: 10.3389/fimmu.2024.1519159 (PMC11790443; doi:10.3389/fimmu.2024.1519159)
Supplement: Supplementary file 3 [file SupplementaryFile1.docx]

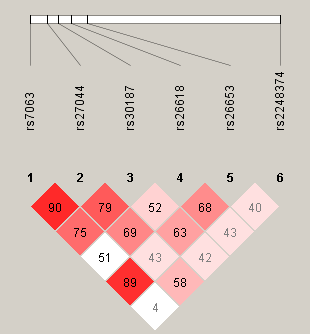

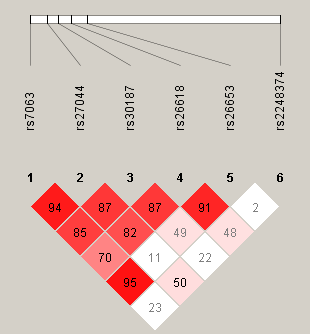


**Supplementary Fig. 1.** LD pattern of the six studied SNPs in the *ERAP1* and *ERAP2* genes in patients (left) and in controls (right). LD was expressed by D’ x 100 value. The darker shading indicates a stronger LD.
